# Degradation of antibiotic resistance genes and mobile gene elements in dairy manure anerobic digestion

**DOI:** 10.1371/journal.pone.0254836

**Published:** 2021-08-25

**Authors:** Yi Wang, Pramod K. Pandey, Sundaram Kuppu, Richard Pereira, Sharif Aly, Ruihong Zhang

**Affiliations:** 1 Department of Population Health and Reproduction, School of Veterinary Medicine, University of California-Davis, Davis, California, United States of America; 2 Department of Biological and Agricultural Engineering, University of California-Davis, Davis, California, United States of America; USDA-ARS Salinity Laboratory, UNITED STATES

## Abstract

Antibiotic resistance genes (ARGs) are emerging contaminants causing serious global health concern. Interventions to address this concern include improving our understanding of methods for treating waste material of human and animal origin that are known to harbor ARGs. Anaerobic digestion is a commonly used process for treating dairy manure, and although effective in reducing ARGs, its mechanism of action is not clear. In this study, we used three ARGs to conducted a longitudinal bench scale anaerobic digestion experiment with various temperatures (28, 36, 44, and 52°C) in triplicate using fresh dairy manure for 30 days to evaluate the reduction of gene abundance. Three ARGs and two mobile genetic elements (MGEs) were studied: sulfonamide resistance gene (*sulII*), tetracycline resistance genes (*tetW*), macrolide-lincosamide-streptogramin B (MLSB) superfamily resistance genes (*erm*F), class 1 integrase gene (*intI1*), and transposase gene (*tnpA*). Genes were quantified by real-time quantitative PCR. Results show that the thermophilic anaerobic digestion (52°C) significantly reduced (*p* < 0.05) the absolute abundance of *sulII* (95%), *intI1* (95%), *tnpA* (77%) and 16S rRNA gene (76%) after 30 days of digestion. A modified Collins–Selleck model was used to fit the decay curve, and results suggest that the gene reduction during the startup phase of anaerobic digestion (first 5 days) was faster than the later stage, and reductions in the first five days were more than 50% for most genes.

## Introduction

Antibiotic resistance is an emerging issue causing serious health concerns. In the United States, at least 2.8 million antibiotic-resistant infections are reported annually, and more than 35,000 human deaths occur as a result of antibiotic-resistant infections [1]. It has been reported that the deaths caused by methicillin-resistant *Staphylococcus aureus* (MRSA) in the US could be higher than that caused by HIV [2,3]. Antimicrobial drugs are known to be important for the selection of antimicrobial resistance in addition to being a shared resource between human and animal populations [4–7].

Each year, more than 11 million kilograms of antimicrobial drugs are used in food-producing animals, within which 52% are medically important drugs [8]. In 2016, 87.5% of US cattle feedlots gave antibiotics in feed, water, or by injection to treat, prevent, and control diseases [9]. More than half of feedlots (55.6%) used medically important antibiotics (i.e., important for treating human disease) in feed. To prevent, control, or treat respiratory disease, 41.8% of feedlots treated cattle with antibiotics in feed. In addition, 80% of feedlots treated sick animals individually with antibiotics by injection.

Up to 90% of used antibiotics are excreted via urine and feces in the unchanged form as well as the active metabolites, including tylosin, tetracycline, and sulfonamides [10,11]. When animal excretion is used as fertilizers on agricultural lands and/or directly deposited on grazing land by livestock, antibiotics and ARGs may be transferred to croplands [12–14]. Excessive use of manure in the field increases the chances of manure borne ARGs in contaminating soil and water resources [12,15]. Some antibiotic resistant bacteria in manure are human pathogens such as *S*. Typhimurium and Shiga-toxigenic *E*. *coli* [16,17], and other bacteria in the environment including pathogens may gain ARGs by horizontal gene transfer (HGT) mechanisms mediated by transposons, integrons, and plasmids [18,19] via transformation, transduction, and conjugation [20,21]. To that end, several national and state regulations to limit the use of antibiotics have been made. For example, the U.S. Drug and Food Administration (FDA)’s directive (Veterinary Feed Directive (VFD) prohibits subtherapeutic doses in animal feed and/or animal drinking water to promote growth and improve feed efficiency. In California, Senate Bill No. 27 (2015) requires a prescription from a California-licensed veterinarian to purchase and use medically important antimicrobial drugs in livestock.

Although policies that limit the uses of antibiotics in animal-agriculture system have the potential to control excessive use of antibiotics, the duration of survival and threat of ARGs being effectively transferred to bacteria in the environment is not understood. Furthermore, relying on natural degradation of ARGs in the environment over time may not be realistic given that studies have shown that the rate of resistance reversibility at the community level may be slow due to evolution, mutations, and genetic co-selection [22,23].

One potential solution for the degradation of ARGs in the environment is anaerobic digestion, which can serve as a step for manure treatment prior to application on crop fields. Previous studies showed anaerobic digestion process could help in reducing ARGs in animal manure [24–27]. Primarily, anaerobic digestion is used to treat manure and produce biogas, a source of renewable energy [28,29]. In addition, anaerobic digestion process produces beneficial organic fertilizer, control odor, and reduces pathogen/bacterial levels in animal waste [30–33]. Compared to mesophilic and ambient-temperature anaerobic process, thermophilic anaerobic process is known to accelerate the digestion process, pathogen reduction, and biogas production [26,34]. Both mesophilic and thermophilic digesters are used to treat organic waste material, however, how an increase in temperature influences ARG removal in dairy manure is not well understood. In general, the impacts of temperature on ARG removal is uncertain [25,35–37].

Hence, the goal of this study was to investigate the effectiveness of anaerobic digestion on neutralizing ARGs in dairy manure. The specific objectives of this research were to: 1) understand the impacts of anaerobic digestion on the reduction of ARGs and MGEs in dairy manure; 2) determine the impacts of mesophilic and thermophilic temperatures on the reduction of ARGs and MGEs; and 3) fit a model to predict gene reductions in anaerobic digestion process over time.

## Materials and methods

### Batch anaerobic digestion experiment of liquid manure

Fresh manure was collected from the University of California, Davis research dairy facility. Manure was diluted with DI water (0.5 L water/kg of manure) and filtered with an 850 μm standard sieve (Fisher Scientific, Hampton, NH). Subsequently, manure was mixed with granules-inocula, as an inoculum to start anaerobic process inside the reactors. The granules were obtained from a mesophilic UASB (i.e., upflow anaerobic sludge blanket) reactor treating potato starch waste (Ingredion Incorporated, Richland, WA).

**Fig 1:**
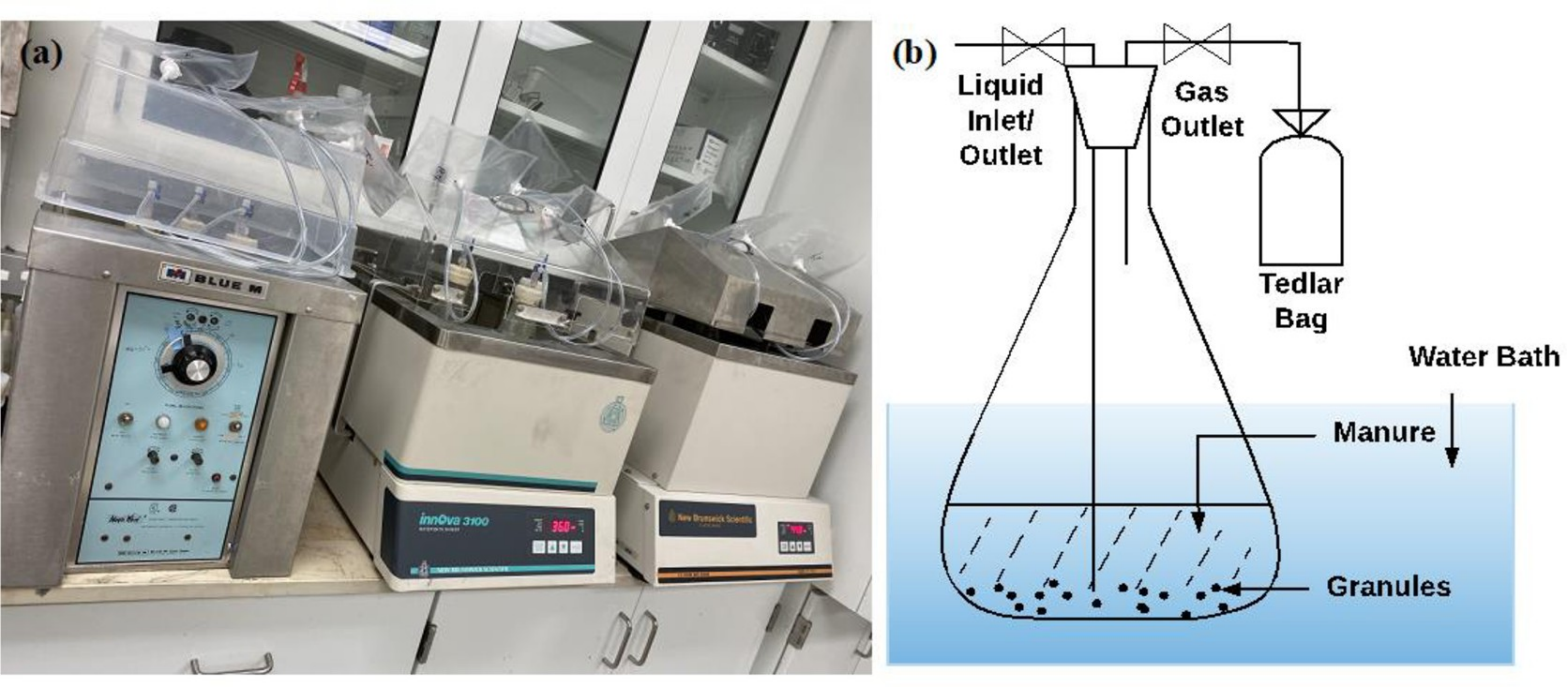
**(a) Anaerobic digester setup. (b) Schematic graph of the anaerobic digester**.

Total solid content (TS) and volatile solid content (VS) of original manure, filtered manure, granules-inocula, and mixed feedstock in this experiment were measured according to the American Public Health Association Mehtods [38]. Mixed feedstock refers to the mixture of granules-inocula and filtered liquid manure that was fed directly to the flask digester. To set up the batch anaerobic digestion experiment (Fig 1), twelve 1000 mL volume Erlenmeyer flasks (Corning Life Sciences, Tewksbury, MA) were filled with 430 mL of liquid manure and 70 g of inocula. Liquid manure and inocula were stirred well before feeding to each flask so that there was no difference between each batch. All the bottles were purged with nitrogen for 2 min and then tightly plugged by a rubber stopper in order to maintain an anaerobic environment. The rubber stopper was inserted by two 8-gauge dispensing needles. One was inserted into the liquid portion for sampling. The other needle in the headspace was connected to a Tedlar bag as a biogas outlet. Connections were sealed by liquid nails (i.e., adhesive) and the seal integrity was tested for each reactor.

The anaerobic digestion batch experiments were performed in four water baths at temperatures 28°C (moderate condition), 36°C (mesophilic condition), 44°C (high mesophilic condition), and 52°C (thermophilic condition). Each temperature had three digesters. Filtered manure, granules-inocula, and mixed feedstock were sampled for DNA extraction and gene quantification. Samples were taken on 0, 1, 2, 3, 5, 7, 10, 14, 18, 22, 26, 30 days, with a total of 12 time points. By connecting a syringe to the liquid inlet, 10 mL digestate at each time point was sampled. After collection, samples were stored at -20°C until use.

### Quantitative reverse transcription PCR (RT-qPCR)

Total DNA was extracted by DNeasy PowerSoil Kit (Qiagen, Valencia, CA). Two mL of each sample was centrifuged at 13,000 rpm for 10 min and the pellet was used for DNA extraction. The quality and concentration of the DNA were assessed by NanoDrop 1000 spectrophotometer (Thermo Scientific, Wilmington, DE). All extracted DNA samples were stored at -20°C before qPCR amplification.

Five types of genes were under specific concern to be investigated in this study: [sulfonamide resistance genes (*sulII*), tetracycline resistance genes (*tetW*), macrolide-lincosamide-streptogramin B (MLS_B_) superfamily (erythromycin is a member) resistance genes (*erm*F), and MGEs (*int*I1, *tnpA*)], which are important for assessing the persistence and dispersal of ARGs. Tetracycline, macrolide, and sulfonamide are three of the most commonly used antibiotics for animal treatment. In 2014, 15.5% of US dairy operations fed tetracycline as medicated milk replacer to preweaned heifers, specifically chlortetracycline (1.6% of operations), Oxytetracycline (4.9% of operations), or combination of neomycin and oxytetracycline (9% of operations) [39]. Furthermore, macrolides were the primary antibiotics to treat respiratory disease in preweaned heifers (18.2% of operations) and weaned heifers (14.1% of operations), and about 66% of weaned heifers were treated by sulfonamides as their primary antibiotics for diarrhea.

Primers designed in previous work were used to amplify targeted ARGs as in Table 1. PCR products were purified, verified by gel electrophoresis, and ligated into a pCR2.1-TOPO vector by TOPO TA Cloning Kit (Invitrogen, Carlsbad, CA) according to the manufacturer’s instructions. Ligations were transformed into DH5α competent cells. Plasmids carrying the targeted ARGs were extracted from cell cultures using QIAprep Spin Miniprep kit (Qiagen, Valencia, CA) and sequenced to verify the insert of the targeted ARGs. The quality and concentration of plasmids were assessed by NanoDrop 1000 spectrophotometer (Thermo Scientific, Wilmington, DE). The copy number of genes per μL plasmid DNA was calculated by [40]:

$$\text{copy}\;\text{of}\;\text{genes}/\mu \text{LDNA}=\frac{b\times c}{L\times a\times 10^{12}}$$ (1)

where *a* is the weight of kb DNA per pmol (1 kb DNA = 0.66 μg/pmol), *b* the Avogadro’s constant (6.022×10^23^/mol), *L* the length of template containing the target gene, *c* the concentration of template measured in μg/μL.

**Table 1:** Synthetic oligonucleotides and temperature regimes used for qPCR reactions.

Primer	Target gene	Sequences (direction 5′–3′)	qPCR annealing temp (°C)	Amplicon size (bp)	Reference
sulII-FW	*sulII*	TCCGGTGGAGGCCGGTATCTGG	60	191	[40]
sulII-RV		CGGGAATGCCATCTGCCTTGAG			
tetW-FW	*tetW*	GAGAGCCTGCTATATGCCAGC	55	168	[41]
tetW-RV		GGGCGTATCCACAATGTTAAC			
ermF-189f	*ermF*	CGACACAGCTTTGGTTGAAC	55	309	[42]
ermF-497r		GGACCTACCTCATAGACAAG			
HS463a	*intI1*	CTGGATTTCGATCACGGCACG	57	473	[43]
HS464		ACATGCGTGTAAATCATCGTCG			
tnpA-04F	*tnpA*-04	CCGATCACGGAAAGCTCAAG	56	101	[44]
tnpA-04R		GGCTCGCATGACTTCGAATC			
357F	16S rRNA gene	CCTACGGGAGGCAGCAG	56	193	[45]
518R		ATTACCGCGGCTGCTGG			

Standard curves of DNA standards were constructed for each 96-well qPCR assay, and each standard curve was generated by 12-fold serial dilutions of plasmid DNA. DNA dilutions that fell out of the linear region of the standard curve were removed from the analysis. Generally, the quantitative limit of qPCR was 10^3^ copies/mL digestate. Efficiencies of PCR standards were calculated by: Efficiency = 10^-(1/slope)^– 1, which were all between 90% and 110% with *r*^2^ ≥ 0.99 for a minimum of six points.

Targeted ARGs and 16S rRNA gene qPCR reactions were performed using the AriaMx Real-time PCR System (Agilent Technologies, Santa Clara, CA) in a 10 μL reaction mixture (5 μL PowerUp SYBR Green Master Mix [2×] (Life Technology, Carlsbad, CA), 0.5 μL 10 mM each primer, and 1 μL of template) with a thermal cycling of 2 min at 50°C for UDG activation and 2 min at 95°C for Dual-Lock™ DNA polymerase activation, followed by 40 cycles of 15 s at 95°C; 15 s at 55°C-60°C; 1 min at 72°C. Each reaction was repeated three times. The average copy and DNA standard deviation were calculated from three dataset for each reaction.

The absolute copy number of genes, which is called absolute abundance (AA), was quantified by referring to the corresponding DNA standard curve obtained by plotting threshold cycles versus log-copy number of genes, which represents gene copy numbers per milliliter of digestate sample. The AA of 16S rRNA gene also represents the total bacterial biomass [46–49]. Levels of targetedARGs were normalized as the percentage of copy number of a gene/copy number of 16S rRNA gene for each sample to emphasize the relative abundance (RA) in manure samples [50–52]. Relative abundance (RA) of genes represents gene absolute abundance normalized to the total number of 16S rRNA genes present in the sample. This normalization provides the proportion of bacterial populations carrying the ARGs/MGEs of interest [53].

### Statistical analysis

Statistical analysis showed gene abundance data followed a log-normal distribution, which was also reported in other studies [47,49]. Therefore, data were log-transformed before statistical analysis. Gene AAs of a total of 144 samples were analyzed, representing 12 reactors at 12 time points. The *erm*F was excluded for further analysis because the gene concentration in samples was below the detection limit (10^3^ copies/mL). Two-factor mixed-design ANOVA analysis was conducted to evaluate the impacts of digestion time and temperature on gene reductions as measured using AA. Each reactor was treated as a block. Time was regarded as a within-subject factor, whereas temperature was a between-subject factor. Residuals passed the Brown-Forsythe test for heteroscedasticity and the Anderson-Darling test for normality (*p* < 0.05). Tukey test was used for multiple comparisons between Day 0, 5, and 30 for each gene (α = 0.05). Multiplicity adjusted *p-*value was reported for each comparison.

Pearson’s correlation matrix between gene relative abundance was generated by pooling of gene abundance data together (144 samples with 12 time points and 12 digesters). The correlation coefficient values range from “0” meaning no correlations to “1” which is a monotone increasing relationship, and “-1” which is a monotone decreasing relationship.

The decay rate of ARGs was determined by a Collins-Selleck model (Eq 2). Collins-Selleck model was initially developed to describe the inactivation of coliform microbes in domestic wastewater by chlorine [54,55]. This model is also used in alternative disinfection methodologies [54]. Previous studies have shown that the decay of ARGs in wastewater solids-amended soil microcosms [49] and anaerobic digestion of municipal wastewater solids [47] can be described by a modified Collins–Selleck model [54,55]. The model (Eq 2) is a power function.


$${\text{log}}_{10}\left(\frac{N}{N_{0}}\right)=\Lambda_{\text{CS}}[{\text{log}}_{10}t-{\text{log}}_{10}b]$$ (2)

In this equation, *N* is the number of ARG copies at time *t*, *N*_*0*_ is the initial quantity of ARGs, *Λ*_*CS*_ is the specific lethality coefficient and *b* is a lag coefficient. Nonlinear regression curves based on gene concentrations versus time was established. The average of three replicates (samples from three digesters) of each treatment (temperature) was used for curve fitting. Least squares regression was chosen as the fitting method. Residuals analysis passed the normality test (Anderson-Darling) and homoscedasticity test checked by the Diagnostics tab. A *p*-value of less than 0.05 was considered significant. All statistical analyses were conducted using GraphPad Prism 8.

## Results and discussion

### Absolute abundance and relative abundance of ARGs and MGEs in anaerobic digestion

The analysis of gene AA in granules-inocula showed *sulII*, *tetW*, *erm*F, *intI1*, and *tnpA* levels were below the detection limit in inoculum, which indicates mixing granules-inocula did not add external ARGs and MGEs of concern into the dairy manure. Total solid content (TS) and volatile solid (VS) mixed feedstocks (filtered liquid manure mixed with granules) were 6.64% and 5.37%, respectively (S1 Table).

**Fig 2:**
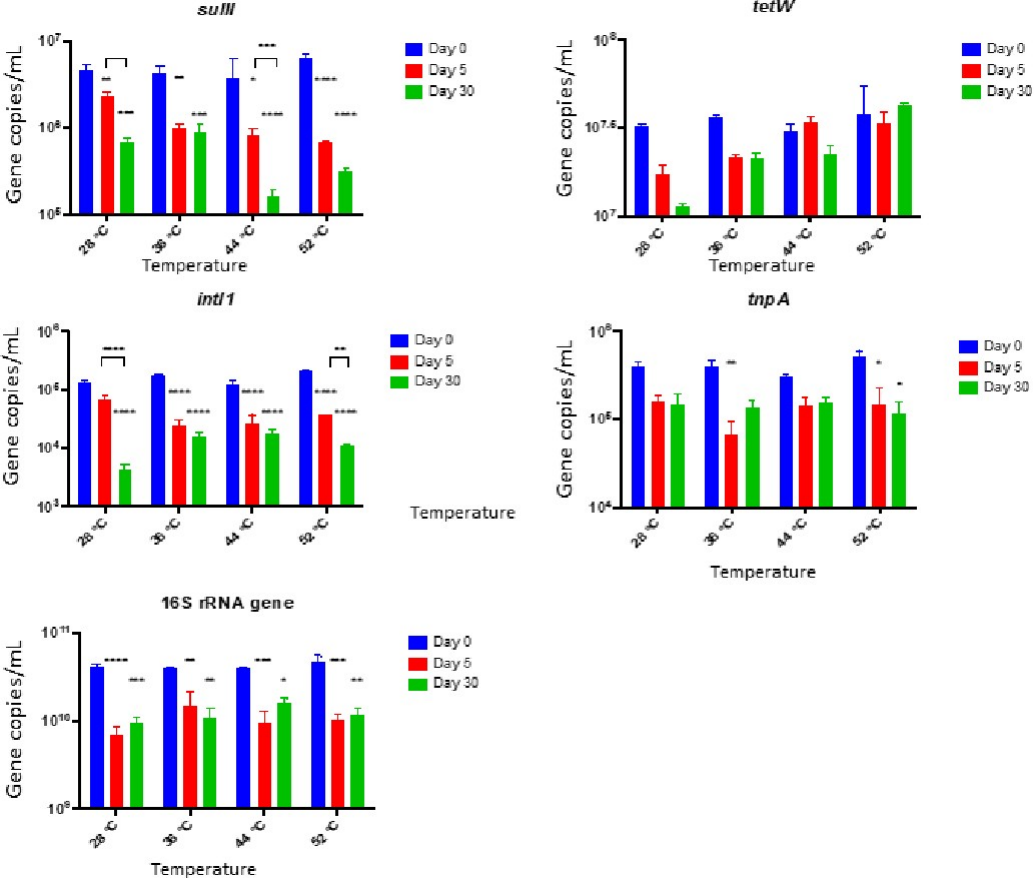
Absolute abundance of ARGs and MGEs at Day 0, 5, 30 in anaerobic digestion. Values represent the means based on three replicates. Stars on bars of Day 5 and Day 30 represent their *p* value when gene AAs were compared with the corresponded initial value (Day 0). Stars on square brackets showed *p* value of comparison between Day 5 and Day 30. Error bars represent standard errors. Symbol and meaning: *p* ≤ 0.05(*); *p* ≤ 0.01(**); *p* ≤ 0.001(***); *p* ≤ 0.0001(****).

Gene absolute abundance at Day 0, 5, 30 were compared as shown in Fig 2. *sulII*, *intI1* and 16S rRNA gene were significantly reduced (*p* < 0.001) in anaerobic digestion at all temperatures by Day 30. *tnpA* was significantly reduced under 52°C (*p* < 0.05) by 77% on Day 30. The reduction in *tetW* abundance was not significant, and *tetW* was increased by 12–13% on Day 5 at 44°C and on Day 30 at 52°C (S2 Table). Results of ANOVA analysis (S3 Table) showed that the impacts of time on gene absolute abundance was significant (*p* ≤ 0.0001). Impacts of temperature and interaction effects of time and temperature on gene *sulII*, *tetW*, *intI1* and 16S rRNA gene were significant (*p* ≤ 0.05). Among all the sources of variation, effect of time explained most of the total variation for each gene, ranging from 43% (*sulII)* to 75% (16S rRNA gene). These results suggest that the impacts of digestion time on reduction of gene AA was considerable during anaerobic digestion process.

At Day 0, *tetW* was the most abundant among targeted ARGs with AA > 10^7^ copies/mL followed by *sulII* (>10^6^ copies/mL). The AAs of both *intI1* and *tnpA* were >10^5^ copies/mL. The AA of 16S rRNA gene were >10^10^ copies/mL. After a digestion of 30 days, the reduction in *sulII* level was significantly higher at 44°C and 52°C compared to 28°C and 36°C (*p* < 0.05). At 44°C and 52°C, *sulII* level was reduced by 95% and 96%, respectively. The *intI1* level at 52°C was reduced by 95%. The highest reduction in *tnpA* was at 52°C (reduced by 77%). However, at low temperature, *tetW* level was reduced by 64% at 28°C.

The reduction in 16S rRNA gene is shown in Fig 2. After 30 days of anerobic digestion, 16S rRNA gene was reduced by 76–77%. In general, *sulII*, *intI1*, *tnpA*, and 16S rRNA gene reductions were overall highest at thermophilic temperature (i.e., 52°C). Similar level of reductions were obtained at 44°C for *sulII* and at 28°C for *intI1* and 16S rRNA. The reduction on gene AA at 52°C was in the following order: *sulII* = *intI1* > *tnpA* = 16S rRNA gene > *tetW*.

While analyzing the impacts of digestion time, results showed that the levels of *sulII* and *intI1* AAs on Day 30 were lower than that of Day 5 under all temperature conditions. This indicates that the longer digestion period will likely to be more effective in reducing genes compared to less than a week of digestion period. The gene reduction percentages from Day 5 to Day 30 was significant in some cases (*sul*II at 28°C and 44°C, *intI1* at 2°C and 52°C). In few genes, for example, the *tnpA* gene level remained at the same level post 5 days of digestion.

**Fig 3:**
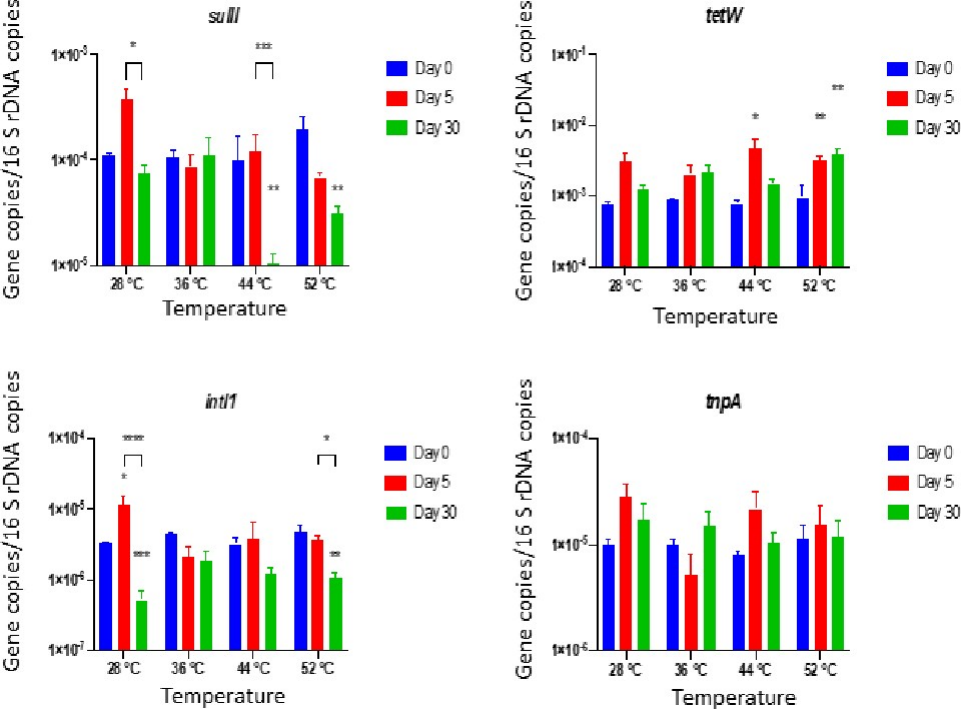
Relative abundance of ARGs and MGEs detected at Day 0, 5, 30 during anaerobic digestion. Values represent the means based on three replicates. Bars represent standard errors. Symbol and meaning: *P* ≤ 0.05(*); *P* ≤ 0.01(**); *P* ≤ 0.001(***); *P* ≤ 0.0001(****).

Relative abundance (i.e., RA) of *sulII*, *intI1* were significantly reduced during anaerobic process (Fig 3). Both 44°C and 52°C resulted in reduction of *sulII* significantly on Day 30 (*p* < 0.01) (S4 Table). Similarly, RA of *intI1* was significantly reduced at 52°C on Day 30 (*p* < 0.01), with reductions of log 0.80 and 0.65, respectively. There were no significant declines in the RAs of *tnpA*. In few cases, for example, ARG *tetW* showed relatively higher level at high mesophilic (44°C) and thermophilic temperature conditions (52°C). In general, RAs of *sulII* and *intI1* were reduced under all temperature conditions at the end of 30 days of anaerobic digestion. In a previous study, a similar anomaly with regards to *tetW* and *tetO* was reported, where Wallace et al. (2018) [56] investigated the abundance of sulfonamides and tetracyclines resistant ARGs (log (ARG Copies/16S rRNA)) in a full-scale anaerobic digester, and authors found that *sul*1 and *sul*2 copies were reduced significantly (*P* < 0.001) by 5% and 10% respectively, however, tetracyclines-related genes (*tetW* and *tetO*) concentrations were unchanged.

Considering the existing information with regards to ARGs reductions in anaerobic digestion process, previous research suggested that anaerobic digestion process may have an impact on reducing ARGs. As an example, Flores Orozco [57] demonstrated that anaerobic digestions removed more than 90% of cephalosporin-resistance gene *cmy-2* in dairy manures in a semi-continuous lab-scale anaerobic digester under mesophilic temperature conditions, with a hydraulic retention time of 30 days. Zhang, Yang [27] found that among 35 studied ARG subtypes, only 8 subtypes were reduced by > 90.0% under thermophilic temperature conditions. A range of other studies, however, suggested that the impact of anaerobic process on gene reduction could be gene specific [25,58–60]. Howes [58] examined changes of antibiotic resistant genes in lab scale anaerobic digesters at thermophilic temperatures, and found that *tetQ* and *cfxA* concentrations reduced significantly by 70%, whereas *mefA* concentrations was increased during 10 days of anaerobic digestion. Similarly, Gao, Gu [61] investigated tetracycline resistance genes and the integrase gene *intI1* in thermophilic anaerobic co-digestion study and reported that all tetracycline resistance genes were reduced except *tetW*. Authors reported that *tetW* encodes ribosomal protection protein, which is relatively more persistent to thermophilic conditions. Besides, the persistence of *tet*W in anaerobic digestion may be explained by a wide variety of microbial hosts. Roberts [62] reported the host range of the *tetW* gene includes Gram-positive, Gram-negative, aerobic and anaerobic bacteria, and *tetW* gene is also associated with conjugative elements which may contribute to its spread. Many factors such as the type of ARGs, bacteria community, digestion parameters, and associated metabolic pathways in the anaerobic digestion process could be attributed to the inconsistent effect of anaerobic digestion on ARG removal, and it also underscores the need for additional research [63].

### Correlations between ARGs and MGEs

**Fig 4:**
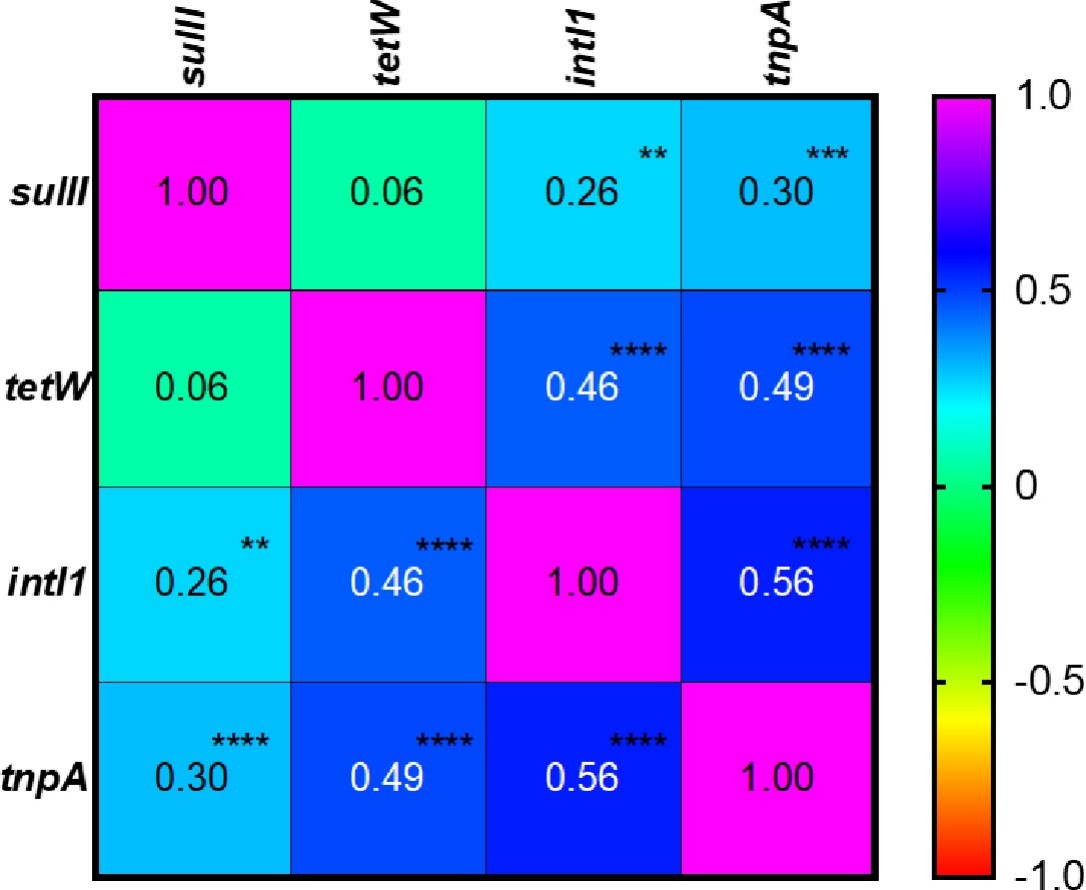
Pearson correlation matrix of relative abundance of ARGs/ MGEs. **significant at *P* ≤ 0.01; *** significant at *P* ≤ 0.001; ****significant at *P* ≤ 0.0001.

Correlation plot of the four genes in Fig 4 showed that *intl1* and *tnpA* were significantly positive correlated with *sulII* and *tetW* (*P* < 0.01). *sulII* and *tetW* were significantly correlated with *intl1*, which means if a sample was found to have high level of *intl1* and *tnpA* abundance, the abundance of *sulII* and *tetW* was higher. The degrees of correlations of *tetW* with *intl1* and *tnpA* were higher than *sulII*, indicating *tetW* abundance was more likely to be positively influenced by *intl1* and *tnpA* abundance than *sulII*. The correlation coefficient of *intl1* and *tnpA* was also significant that indicates that *intl1* tended to co-exist with *tnpA*. The correlation coefficient of *sulII* and *tetW* was found to be low (-0.01) and not significant (*p* = 0.89), which indicates that the abundance of these genes was not correlated. Interspecies gene transfer may have played a role up to an extent to establish linkages between genes. Sørensen, Bailey [64] suggested horizontal gene transfer (due to gene transfer via MGEs between different bacterial hosts) and vertical gene transfer (due to the proliferation of bacterial hosts) are two main factors for ARGs abundance. Mobile genetic elements (MGEs), including transposons, plasmids, and integrons facilitates horizontal gene transfer (HGT) and induce a higher abundance of ARGs [44]. Mobile integrons usually carry ARGs and mobility is supported by transposons and plasmids [65]. Huang, Zheng [35] investigated transposase genes and ARGs during anaerobic digestion of swine manure, and transposase genes showed significant correlations with most ARG types including sulfonamide and tetracycline resistance genes.

### Gene reduction curves fit using Collins-Selleck model

**Fig 5:**
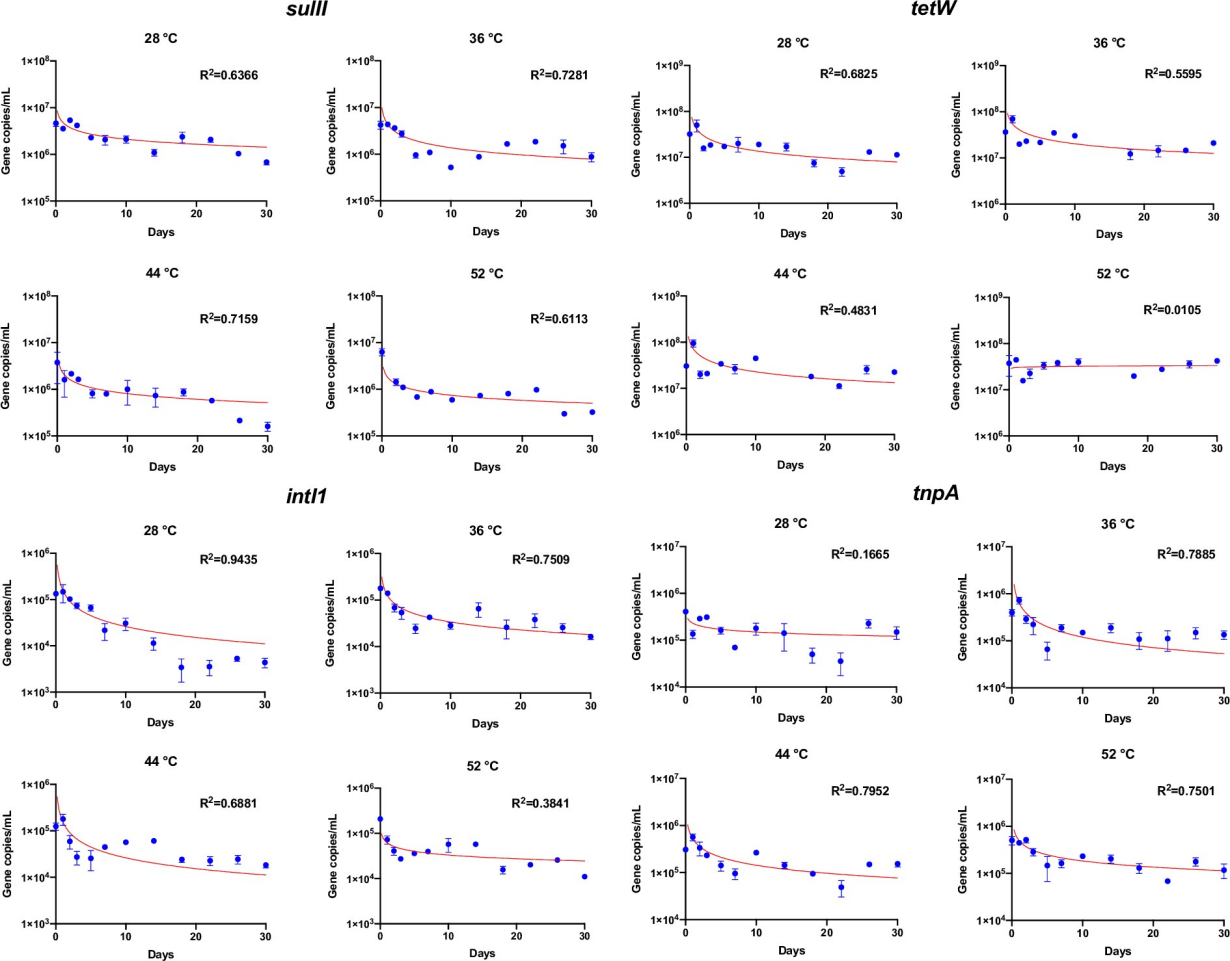
Modelling fit of *sulII*, *tetW*, *intI1*, and *tnpA*. Bars represent standard errors. Blue dots represent the mean value of triplicate reactors under each temperature. Solid red lines represent the best fit of the data to a modified Collins–Selleck model. The model coefficients are shown in Table 2.

**Fig 6:**
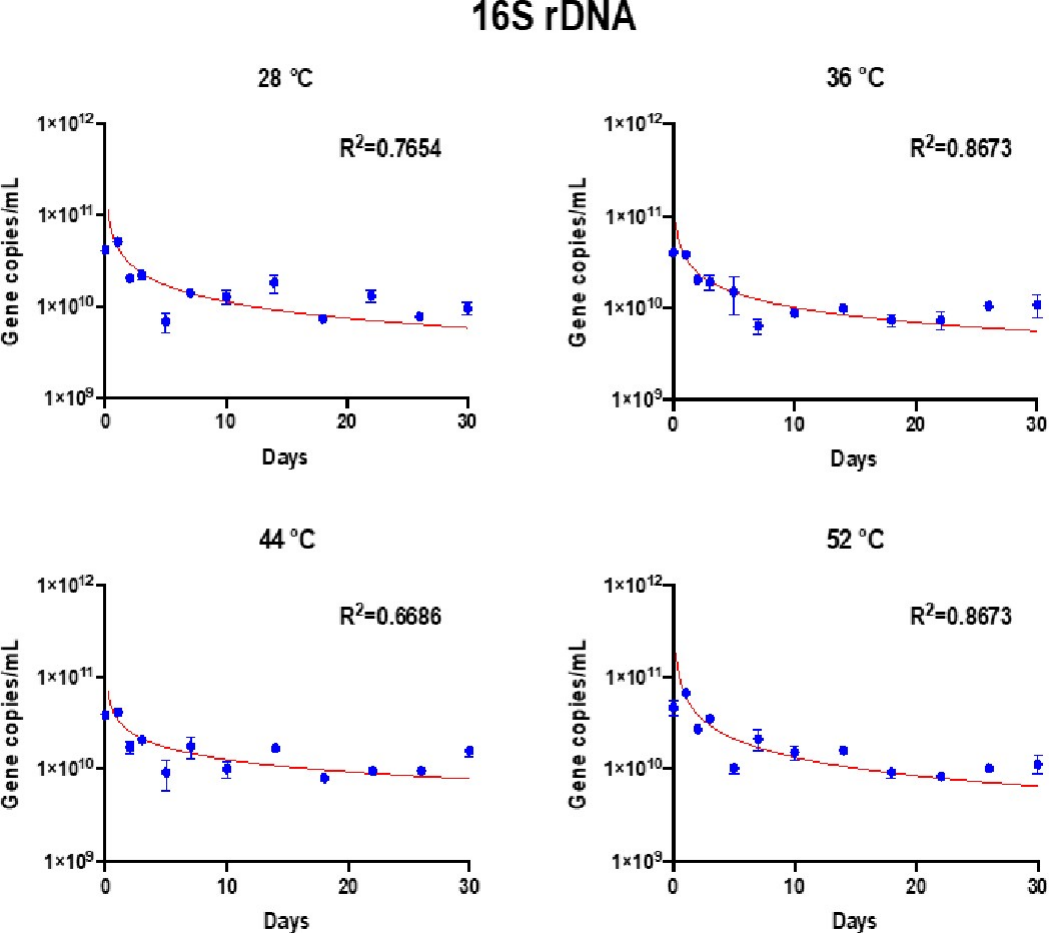
16S rRNA gene in anaerobic digestion over time. Bars represent standard errors. Blue dots represent the mean value of triplicate reactors under each temperature.

Results showed that the modified Collins–Selleck model provided a good fit for estimating the reductions of ARGs, MGEs, and 16S rRNA genes over time during the anaerobic digestion (Figs 5 and 6) except *tetW* and *tnpA*, where *r*^*2*^ value was low. The low *r*^*2*^ value was caused by increase of the gene abundance over time. The coefficients of model fit are shown in Table 2.

**Table 2:** Coefficients for modified Collins–Selleck model by least squares nonlinear regression.

Targeted gene	Temperature	*N*_*0*_	*Λ*_*CS*_	*B*	*R* ^*2*^	Reduction at Day 5
*sulII*	28°C	4.62E+06	-0.36	1.12	0.64	41%
	36°C	4.26E+06	-0.50	1.07	0.73	54%
	44°C	3.77E+06	-0.41	0.23	0.72	72%
	52°C	6.30E+06	-0.35	0.02	0.61	85%
*tetW*	28°C	3.21E+07	-0.49	1.70	0.68	41%
	36°C	3.63E+07	-0.43	2.62	0.56	24%
	44°C	3.04E+07	-0.50	5.67	0.48	-6%
	*52°C*	*3*.*75E+07*	*0*.*03*	*1716*.*11*	*0*.*01*	*15%*
*intI1*	28°C	1.34E+05	-0.78	1.24	0.94	66%
	36°C	1.78E+05	-0.57	0.53	0.75	72%
	44°C	1.25E+05	-0.77	1.33	0.69	64%
	52°C	2.09E+05	-0.27	0.01	0.38	81%
*tnpA*	*28°C*	*4*.*09E+05*	*-0*.*19*	*0*.*05*	*0*.*17*	*58%*
	36°C	4.00E+05	-0.74	1.94	0.79	50%
	44°C	3.07E+05	-0.57	2.59	0.80	31%
	52°C	5.03E+05	-0.43	0.98	0.75	51%
16S rRNA gene	28°C	4.13E+10	-0.60	1.14	0.77	59%
	36°C	4.00E+10	-0.54	0.80	0.87	63%
	44°C	3.91E+10	-0.44	0.80	0.67	55%
	52°C	4.70E+10	-0.67	1.55	0.87	54%

*N*_*0*_ is the initial quantity of genes (copies/mL) at Day 0. *Λ*_*CS*_ is the specific lethality coefficient (unitless) and *b* is a lag coefficient (copies*day/mL). *R*^*2*^ indicates goodness of fit. Normality of Residuals were checked by Anderson-Darling test. Model also passed homoscedasticity test.

*Λ*_*CS*_ was the slope of the transformed log X and Y curve, which described the rate of reduction for each gene. At 36°C, *sulII* gene reduction rate was 1–2 times greater than that at 28°C and 52°C. *tnpA* reduction rate was also higher at temperature 36°C. For *tetW* and *intI1*, however, the reduction rate was found to be greater at 28°C and 44°C. 16S rRNA gene reduction rate was highest at 52°C.

It is important to note that this study uses SYBR green approach for evaluating ARGs. Other alternative methods such as TaqMan probe provides improved specificity. There are advantages and disadvantages in both methods (TaqMan and SYBR). While SYBR is easy and cost effective, specificity is better in TaqMan, however, TaqMan requires specific probe for each target. Considering the important issues such as ARGs, it is important to adapt multiple approaches and testing methods for robust dataset in order to make the informed decision and reduce the biasness caused by testing methods. When grouping coefficients by temperatures, there was no significant difference among the specific lethality coefficient *Λ*_*CS*_ under different temperature conditions, which indicates that the decay rate of each gene might vary with temperature. The lethality coefficient could be gene specific. The *intI1*, *tnpA*, and 16S rRNA genes had higher reduction rates (i.e., specific lethality coefficient *Λ*_*CS*_ compared to *sulII* and *tetW* (*P* < 0.05). In most cases, ARGs/MGEs declined quickly in the beginning but then the reduction slowed down. Reductions by Day 5 were generally above 50% for all the genes except *tetW* (Table 2). The quick reduction of targeted ARGs in the beginning phase of anaerobic digestion could be explained by the significant reduction of aerobic bacteria such as Actinomycetales and Bacilli [66]. The calculations of reductions rates using the modeling tools such as Collins–Selleck can provide insights while comparing the impacts of various treatment on gene reductions, and help in decision making in terms of temperature and retention time needed for controlling various genes in digestate.

The use of Collins-Selleck model for understanding the decay of ARGs in landfill leachate in aerobic and anaerobic conditions [67], land application of sludge bio-drying products [68], and thermophilic aerobic digestion of sewage sludge [69] are reported and results indicate that this model can assist in understanding the reduction rates and determining the impacts of environmental conditions on ARGs reductions. In a previous study, Burch, Sadowsky [70] investigated the fate of six ARGs (*sul*1, *tet*A, *tetW*, *tet*X, *erm*B, *qnr*A) and one integrase gene (*intI1*) during various treatments conditions (air drying, aerobic digestion, mesophilic/ thermophilic anaerobic digestion, pasteurization, and alkaline stabilization) of wastewater solids-amended soil microcosms. The authors reported that in most cases, ARGs declined quickly in the initial phase of treatment, and subsequently the reduction rate was reduced. The model was able to capture the reduction rates under various conditions. Similarly, another study by Burch, Sadowsky [47] conducted laboratory-scale thermophilic anaerobic digestion experiments at 40, 56, 60, and 63°C. The authors reported decrease in ARGs (*tetW*, *tet*X, *qnr*A) and *intI1* abundance under all temperature conditions, and reduction rates of genes could be understood better by Collins-Selleck model than first-order and second-order kinetic models. Sandberg and LaPara [49] investigated the fate of ARGs (*erm*B, *tet*A, *tetW*, *tet*X) and class 1 integron (*intI1*) in swine and dairy manure amended soil and found that Collins–Selleck model described the decay of ARGs in the soil microcosms well. Other methods such as linear model fit may underestimate the length of time needed for ARG reduction.

While there are previous studies elaborating the impacts of anaerobic digestion of ARG reductions [71–75] in a range of feedstocks such as waste water, sludge and dairy manure, the performance of anaerobic digesters substantially depends on the feedstock, therefore, it is important to consider existing manure management in animal-agriculture system and the type of feedstock fed to digesters in order to understand the possible impacts of anaerobic digestion on ARGs reductions. For example, study by Zhang et al. (2011) [71] is focused on studying activated sludge, and sludge characteristics differ than flushed manure. Similarly, research by Ma et al. (2011) [72] studied waste water treatment process of sludge in thermophilic temperature. While the principles of anaerobic digestions process are similar but feedstock characteristics have substantial impacts on anaerobic process, which may be one possible reason for differences in results among studies. Antunes [75] reported that sulfonamide resistance genes are likely to be correlated with class 1 integron. Article by Sun et al (2016) [73] studied scrapped dairy manure in moderate, mesophilic, and thermophilic temperature to understand the dynamics and bacterial communities by q PCR and 16S rRNA and results indicate that anaerobic digestion has a potential to influence ARGs. Temperature can cause selective selection of bacteria carrying a lower number of ARG on their genome plasmids, which can help in ARGs removal. This study was focused on evaluating the manure of flush system, which is often used for flushing the manure from confined dairy operations, and the results of this study may not be transferable to sludge digester systems. Additional studies are needed to improve the existing knowledge for various types of organic matter fed to anaerobic digesters. As antibiotic concern is relatively a new issue, existing set of knowledge corresponding to various waste treatment processes and feedstock characteristics yet to be developed robustly. Future studies, and existing knowledge likely to help in developing improved treatment methods, which can likely to reduce the possible ARGs load from waste material to environment.

## Conclusions

This study was focused on evaluating anaerobic digesters impacts on ARGs removal in dairy manure management system, where flush system was used. Findings of this study based on three ARGs, and 16S rRNA gene indicates that anaerobic digesters can help reducing ARGs in dairy manure, however, additional studies are needed to improve the findings, which elaborates further on the effects of feedstock, and temperature. Results showed that after 30-day thermophilic digestion (52°C), the absolute abundance of *sulII*, *intI1*, *tnpA*, 16S rRNA gene was reduced by 76%-95%, however, absolute abundance of *tetW* was increased by 13%. Reduction of *intI1* and 16S rRNA was comparable at moderate and thermophilic conditions, indicating that the impact of thermophilic anaerobic digestion on gene reduction may not necessarily be greater than lower temperatures. Results of decay curves fitted with modified Collins–Selleck model indicates that reduction of antibiotic resistance genes during startup phase was greater than the later stages, when dairy manure inside digester was relatively acclimatized.

## Supporting information

S1 Table TS, VS and VS/TS of original manure, filtered manure, granules-inocula, and mixed feedstock. (DOCX)

S2 Table Gene AA reductions on Day 5 and Day 30 compared with Day 0. Numbers are in percentage.
(DOCX)

S3 Table Two-factor mixed-design ANOVA for effects of time and temperature on ARGs. (DOCX)

S4 Table Gene RA reductions on Day 5 and Day 30 compared with Day 0. Numbers are in log scale.
(DOCX)
