# Supplementary material for: Degradation of antibiotic resistance genes and mobile gene elements in dairy manure anerobic digestion
Source: PLoS One. 2021 Aug 25;16(8):e0254836. doi: 10.1371/journal.pone.0254836 (PMC8386849; doi:10.1371/journal.pone.0254836)
Supplement: S1 Table — (DOCX) [file pone.0254836.s001.docx]

S1 Table. TS, VS and VS/TS of original manure, filtered manure, granules-inocula, and mixed feedstock

|  | TS | VS | VS/TS |
| --- | --- | --- | --- |
| Original manure | 14.43% | 12.24% | 84.84% |
| Filtered manure | 5.75% | 4.50% | 78.16% |
| Granules-inocula | 13.13% | 11.55% | 87.94% |
| Mixed feedstock | 6.64% | 5.37% | 80.90% |
