# Supplementary material for: Degradation of antibiotic resistance genes and mobile gene elements in dairy manure anerobic digestion
Source: PLoS One. 2021 Aug 25;16(8):e0254836. doi: 10.1371/journal.pone.0254836 (PMC8386849; doi:10.1371/journal.pone.0254836)
Supplement: S2 Table — Numbers are in percentage. (DOCX) [file pone.0254836.s002.docx]

S2 Table. Gene AA reductions on Day 5 and Day 30 compared with Day 0. Numbers are in percentage.

*Significant at *P* ≤ 0.05, **Significant at *P* ≤ 0.01, ***Significant at *P* ≤ 0.001, ****Significant at *P* ≤ 0.0001.

|  | *sulII* | | *tetW* | | *intI1* | | *tnpA* | | 16S rRNA gene | |
| --- | --- | --- | --- | --- | --- | --- | --- | --- | --- | --- |
|  | Day 5 | Day 30 | Day 5 | Day 30 | Day 5 | Day 30 | Day 5 | Day 30 | Day 5 | Day 30 |
| 28 °C | -51% | -85%*** | -46% | -64% | -50% | -97%**** | -60% | -64% | -83%**** | -77%*** |
| 36 °C | -77%** | -79%*** | -40% | -42% | -86%**** | -91%**** | -83%** | -66% | -63%** | -73%** |
| 44 °C | -78%* | -96%**** | 12% | -26% | -79%**** | -85%**** | -54% | -50% | -76%*** | -59%* |
| 52 °C | -89%**** | -95%**** | -10% | 13% | -83%**** | -95%**** | -71%* | -77%* | -78%*** | -76%** |
