# Supplementary material for: Degradation of antibiotic resistance genes and mobile gene elements in dairy manure anerobic digestion
Source: PLoS One. 2021 Aug 25;16(8):e0254836. doi: 10.1371/journal.pone.0254836 (PMC8386849; doi:10.1371/journal.pone.0254836)
Supplement: S3 Table — (DOCX) [file pone.0254836.s003.docx]

S3 Table. Two-factor mixed-design ANOVA for effects of time and temperature on ARGs

|  | *sulII* | | *tetW* | | *intI1* | | *tnpA* | | 16S rRNA gene | |
| --- | --- | --- | --- | --- | --- | --- | --- | --- | --- | --- |
| Source of Variation | % of total variation | P value | % of total variation | P value | % of total variation | P value | % of total variation | P value | % of total variation | P value |
| Time $\times$  Temperature (interaction effect) | 20.00 | <0.0001 | 16.74 | 0.0030 | 21.99 | <0.0001 | 12.94 | 0.1464 | 8.305 | 0.0053 |
| Time | 43.02 | <0.0001 | 44.68 | <0.0001 | 59.07 | <0.0001 | 53.61 | <0.0001 | 75.87 | <0.0001 |
| Temperature | 20.31 | 0.0005 | 15.75 | 0.0002 | 7.061 | 0.0064 | 1.690 | 0.5413 | 3.454 | 0.0130 |
| Subject | 2.733 | 0.0386 | 1.670 | 0.5462 | 2.131 | 0.0214 | 5.836 | 0.0180 | 1.332 | 0.2411 |
