# Supplementary material for: Degradation of antibiotic resistance genes and mobile gene elements in dairy manure anerobic digestion
Source: PLoS One. 2021 Aug 25;16(8):e0254836. doi: 10.1371/journal.pone.0254836 (PMC8386849; doi:10.1371/journal.pone.0254836)
Supplement: S4 Table — Numbers are in log scale. (DOCX) [file pone.0254836.s004.docx]

S4 Table. Gene RA reductions on Day 5 and Day 30 compared with Day 0. Numbers are in log scale.

*Significant at *P* ≤ 0.05, **Significant at *P* ≤ 0.01, ***Significant at *P* ≤ 0.001.

|  | *sulII* | | *tetW* | | *intI1* | | *tnpA* | |
| --- | --- | --- | --- | --- | --- | --- | --- | --- |
|  | Day 5 | Day 30 | Day 5 | Day 30 | Day 5 | Day 30 | Day 5 | Day 30 |
| 28 °C | 0.53 | -0.17 | 0.59 | 0.20 | 0.55* | -0.80*** | 0.45 | 0.23 |
| 36 °C | -0.09 | 0.02 | 0.35 | 0.39 | -0.31 | -0.38 | -0.28 | 0.18 |
| 44 °C | 0.08 | -0.98** | 0.79* | 0.28 | 0.09 | -0.42 | 0.44 | 0.12 |
| 52 °C | -0.46 | -0.80** | 0.53** | 0.63** | -0.12 | -0.65** | 0.12 | 0.01 |
